# Impact of Temperature on Low-Cycle Fatigue Characteristics of the HR6W Alloy

**DOI:** 10.3390/ma14226741

**Published:** 2021-11-09

**Authors:** Grzegorz Junak, Anżelina Marek, Michał Paduchowicz

**Affiliations:** 1 Faculty of Materials Engineering, Silesian University of Technology, 40-019 Katowice, Poland; anzelina.marek@polsl.pl; 2 Faculty of Mechanical Engineering, Wrocław University of Science and Technology, 50-370 Wrocław, Poland; michal.paduchowicz@pwr.edu.pl

**Keywords:** HR6W alloy, low-cycle fatigue, cyclic strengthening curve, fatigue life

## Abstract

This paper presents the results of tests conducted on the HR6W (23Cr-45Ni-6W-Nb-Ti-B) alloy under low-cycle fatigue at room temperature and at 650 °C. Fatigue tests were carried out at constant values of the total strain ranges. The alloy under low-cycle fatigue showed cyclic strengthening both at room temperature and at 650 °C. The degree of HR6W strengthening described by coefficient n’ was higher at higher temperatures. At the same time, its fatigue life N_f_ at room temperature was, depending on the range of total strain adopted in the tests, several times higher than observed at 650 °C.

## 1. Introduction

In Poland, electricity is mainly produced in conventional power plants, i.e., those in which the steam needed to drive the generators comes from coal-fired or lignite-fired boilers. These power plants emit significant amounts of CO_2_ to the atmosphere. With European policy aiming to reduce CO_2_ emissions into the atmosphere, the Polish energy sector needs to adapt to European regulations. The reduction in CO_2_ emissions can be achieved, among others, by increasing the operating parameters of supercritical boilers with constantly improved advanced materials which have better properties at elevated temperatures [1,2,3,4,5,6,7]. Such materials include the HR6W alloy, which is mainly used for superheater tubes in power boilers, thick-walled tubular elements, reactors, as well as in chemical plants.

What is required from materials under high temperatures and pressures, which occur in boilers under increased operating parameters, is high creep resistance and thermal-mechanical and low-cycle fatigue resistance as well as high corrosion resistance [1,2,3,4,5,6,7,8,9,10,11,12,13,14,15,16,17,18,19,20,21,22]. Unfortunately, in contrast to the large number of generally available data in the field of research on basic mechanical properties at room temperature and elevated temperature, creep, and corrosion resistance of the HR6W alloy [1,2,3,4,5,6,7,8,9,10,11,12,13,15,16,17,18,19], there is less data on thermo-mechanical and low-cycle fatigue testing of this alloy [2,6,14,20]. The benefits of using data obtained in low-cycle fatigue tests at elevated temperature for prediction of durability under thermo-mechanical fatigue loading for the HR6W alloy were demonstrated by Noguchi et al. [20]. The authors of the paper [20] investigated the relationship between the fatigue life of HR6W in isothermal fatigue tests and the fatigue life obtained in thermo-mechanical fatigue tests. The tests were carried out in the temperature range between 100 and 700 °C. Researchers pointed out a similarity between these types of tests for this alloy. Therefore, it seems justified to estimate the strength of the alloy under thermo-mechanical fatigue based on less complex, more feasible isothermal fatigue tests. In the work presented, a study of low-cycle fatigue at room temperature and at 650 °C constituted a primary research experiment. This research is meant as a contribution to the existing knowledge on the cyclic behavior of the HR6W alloy with the properties determined at 650 °C. Therefore, the test results obtained can be useful for more precise forecasting of the fatigue life of power plant components.

## 2. Materials and Methods 

**Figure 1:**
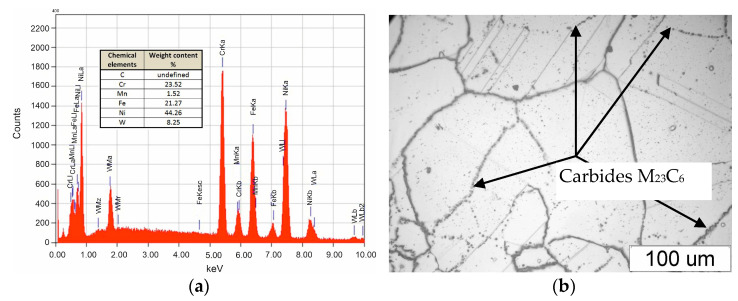
SEM. X-ray energy spectrum of the as-delivered HR6W (**a**); microstructure of the as-delivered HR6W alloy (**b**).

The material for low-cycle fatigue testing was the as-delivered HR6W (23Cr-45Ni-6W-Nb-Ti-B) alloy taken from a fragment of a thick-walled pipe. The chemical composition of the alloy was determined on the basis of the X-ray energy spectrum as presented in Figure 1a. A microanalysis of the chemical composition of the alloy was performed using the EDS Oxford LINK ISIS-300 X-ray (Manufacture: Oxford Instruments plc) microanalyzer coupled with a JEOL JSM 5800 LV scanning electron microscope (Manufacture: Oxford Instruments plc).

In the as-delivered condition, the material has an austenite structure, which is characterized by a non-uniform grain size. Numerous precipitations of M_23_C_6_ carbides were observed at the grain boundaries (Figure 1b).

**Figure 2:**
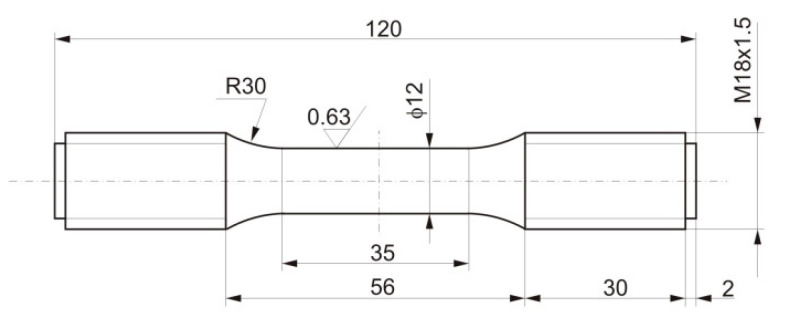
Dimensions in millimeters of a HR6W sample for low-cycle fatigue testing.

Fatigue test samples (Figure 2) were made of material taken from the ϕ360 mm × 75 mm pipe.

Mechanical properties were tested at room temperature and at 650 °C. The test results are presented in Table 1.

**Figure 3:**
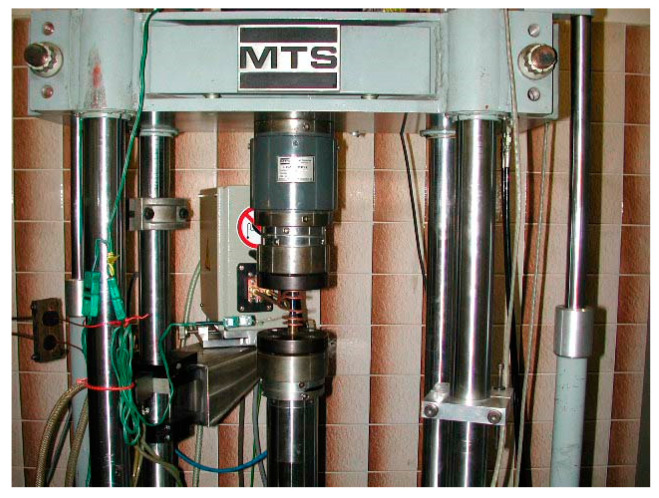
MTS-810 testing machine used in low-cycle testing.

**Figure 4:**
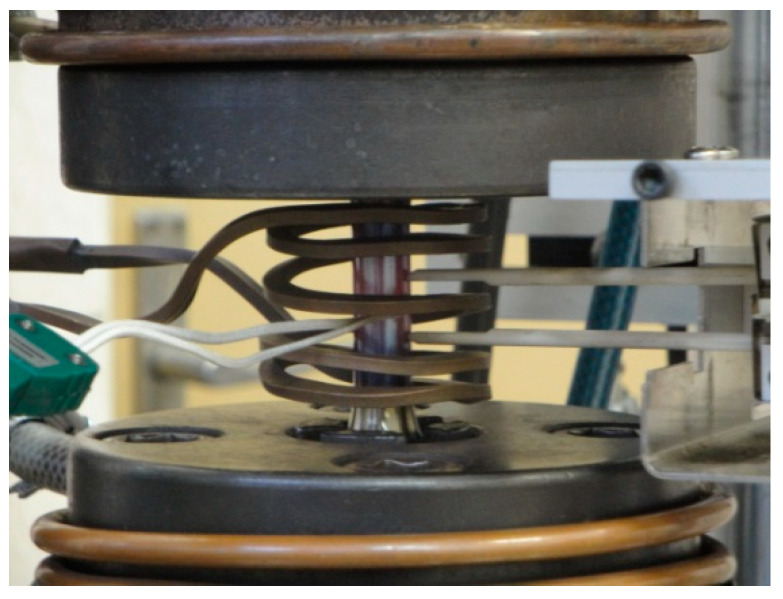
Testing station for low-cycle fatigue tests at 650 °C.

Fatigue tests for the low-cycle HR6W alloy were carried out on the MTS-810 testing machine (Figure 3).The tests were run in strain control (extensometer MTS-632-11C-20 temp. room, extensometer MTS-632-14B-05 temp. 650 °C). Sinusoidal loading cycles were applied with a cycle asymmetry ratio R = −1 and load frequency f = 0.1 Hz. The fatigue tests were carried out at room temperature and at 650 °C. The tests were carried out for five ranges of total deformation Δε_t_ = 0.6; 0.7; 0.8; 1.0 and 1.2%. The samples were induction heated using a Hüttinger TIG-300 heater (Figure 4). Low-cycle fatigue tests at elevated temperatures were carried out with the use of a FLIR SC6000 thermal imaging camera, which allowed continuous temperature control during the tests.

## 3. Results and Discussion

**Figure 5:**
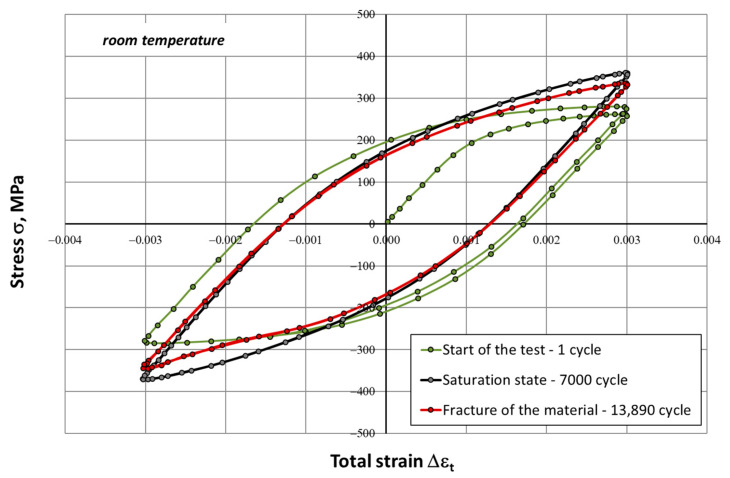
Hysteresis loops determined in a low-cycle fatigue test at room temperature for the strain range ∆ε_t_ = 0.6%.

**Figure 6:**
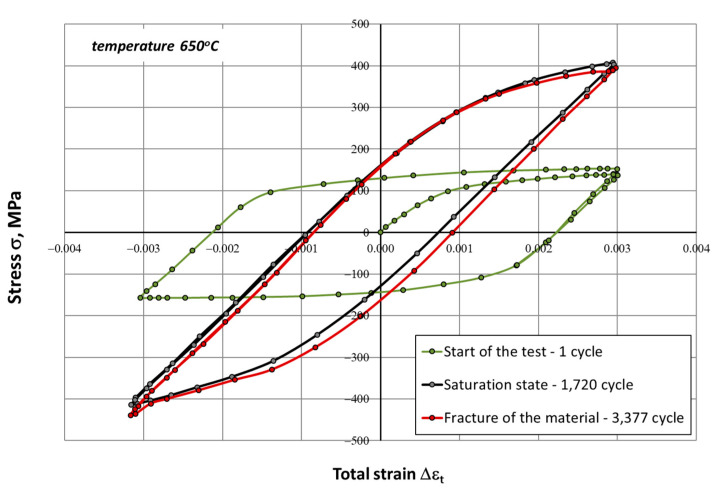
Hysteresis loops determined in a low-cycle fatigue test at 650 °C for the strain range ∆ε_t_ = 0.6%.

During the fatigue tests, changes in stress amplitude (σ_an_) and strain (ε_t_) versus the number of cycles (N) were constantly recorded. On this basis, hysteresis loops characteristic for selected stages of the fatigue process were developed, i.e., the initial phase, the saturation phase (characterized by a fixed value of the stress amplitude σ_an_), and the fracture phase for the sample. Examples of such graphs for the selected strain range ∆ε_t_ = 0.6% are shown in Figure 5 and Figure 6. In low-cycle tests, fatigue life N_f_ was defined as the number of cycles to failure for the sample.

**Figure 7:**
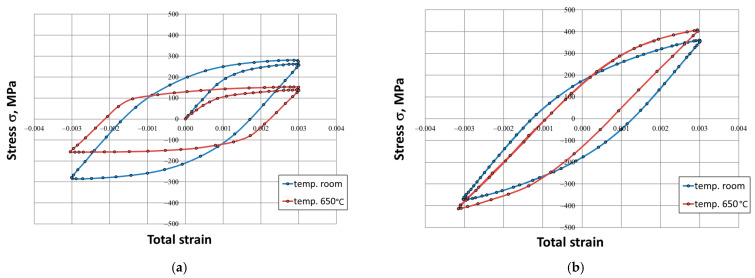
Comparison of the hysteresis loops determined in the low-cycle fatigue test at room temperature and at 650 °C for the strain range ∆ε_t_ = 0.6%: initial state—(**a**), saturation state—(**b**).

The process of cyclic hardening of the HR6W alloy is clearly visible in the above hysteresis loops obtained in low-cycle fatigue tests at room temperature and at elevated temperature (650 °C) for the total strain range of 0.6%. It is manifested in an increase between the stress values read at the tops of the hysteresis loop corresponding to the beginning of the test and the saturation state. A particularly notable amplification occurs for samples tested at 650 °C, where the stress value increases more than twice, from a level of about 160 MPa for the initial loop to 400 MPa corresponding to the saturation loop. Moreover, the saturation stresses recorded in the tests carried out at 650 °C were higher than those recorded at room temperature (Figure 7b).

Both at room temperature and at elevated temperature, a distinct change in the shape of the hysteresis loop was observed (Figure 5, Figure 6 and Figure 7), which is linked directly with material hardening in the process of cyclic deformation. The shape and the dimensions of the hysteresis loop indicated a very large increase in the maximum and the minimum stress. This was especially evident at elevated temperature (650 °C). Cyclic plastic strains occurring in the internal structure of the material may also cause different stress states in differently-oriented grains. This can trigger plastic deformation mechanisms, which results in the Bauschinger effect. This effect is caused by both mechanical strengthening, presumably related to the reconstruction of dislocation systems and the increase in their density, as well as to the precipitation processes caused by prolonged exposure to elevated temperature.

Such behavior of the HR6W alloy is linked to precipitation strengthening occurring at its grain boundaries, responsible for blocking the slip bands. In the case of this alloy, precipitation processes are intensified as a result of high temperatures. Therefore, in order to determine the reasons for the hardening of the HR6W alloy at 650 °C, metallographic tests of the material taken from the samples submitted to the low-cycle fatigue tests were carried out. 

It can also be seen that the maximum stress in the first load cycle at room temperature is higher than the maximum stress in the first cycle at 650 °C. This behavior of the material which differs in the saturated state in relation to the first load cycle results from the simultaneous impact of the temperature and the number of load cycles on the strengthening process. On the one hand, an increased temperature affects the weakening of the material; on the other hand, its long-term effect favors precipitation processes that lead to strengthening [12].

The changed behavior of the material in the saturated state results from the simultaneous influence of the temperature and the number of load cycles on the material strengthening process. On the one hand, the increase in temperature affects the weakening of the material, yet, on the other, its long-term effect favors precipitation processes, thus leading to the strengthening of the material. 

**Figure 8:**
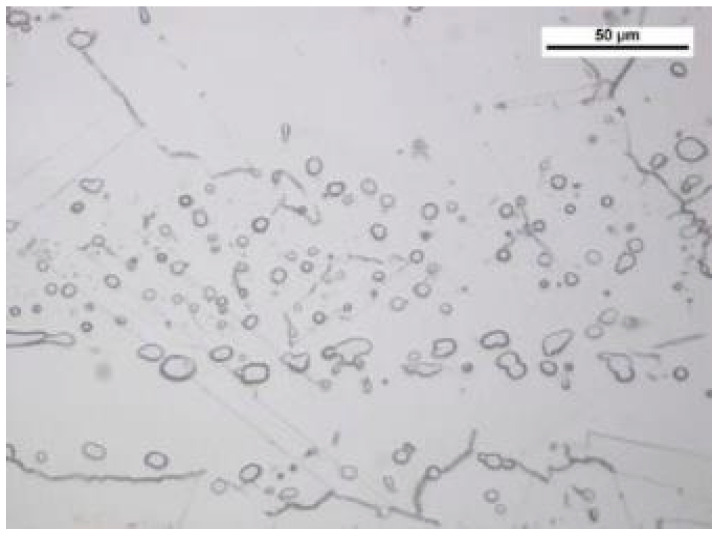
High-nickel austenite with banded carbide precipitates M_23_C_6_. Light microscopy. Mi30Fe, a sample material subjected to a total strain of 0.6% at 650 °C, was etched.

**Figure 9:**
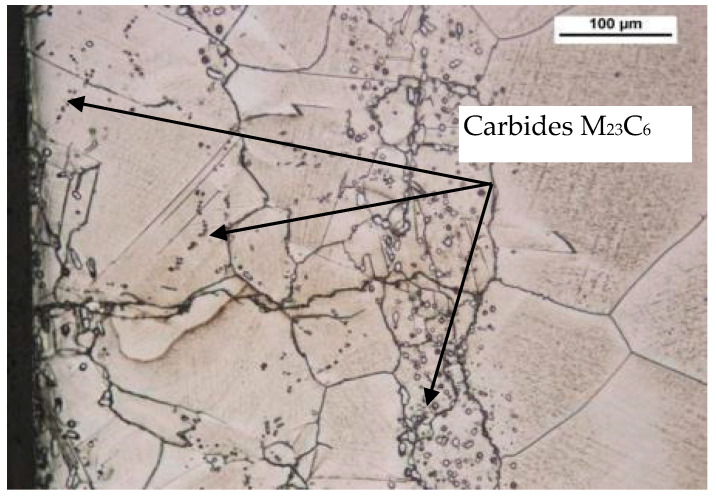
Microstructure of the HR6W alloy. Light microscopy Mi30Fe, a sample material subjected to a total strain of 0.7% at 650 °C, was etched.

Figure 8 and Figure 9 present the metallographic structures obtained for the samples under observation.

Based on the analysis of the obtained specimens, coarse-grained, high-nickel austenite with visible twins and streaked precipitates of carbides M_23_C_6_ was found. These precipitates were distributed in the direction of plastic forming. Carbides M_23_C_6_ distributed on the boundaries of austenite grains create a local continuous shells. In addition, this alloy was shown to form both MC carbides. All these structural precipitates are in turn responsible for such strong strengthening of the HR6W material as observed in low-cycle fatigue testing at elevated temperature.

**Figure 10:**
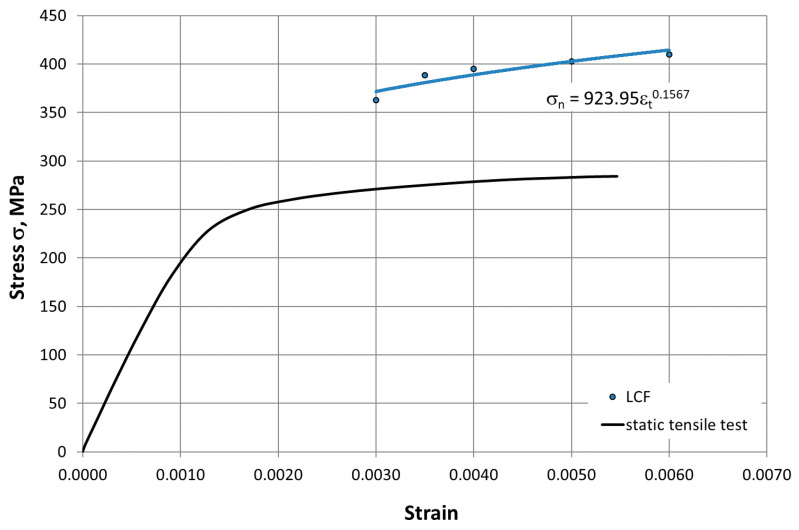
Cyclic strengthening curve for HR6W alloy at room temperature against a curve determined in a static tensile test (LCF—Low Cycle Fatigue).

**Figure 11:**
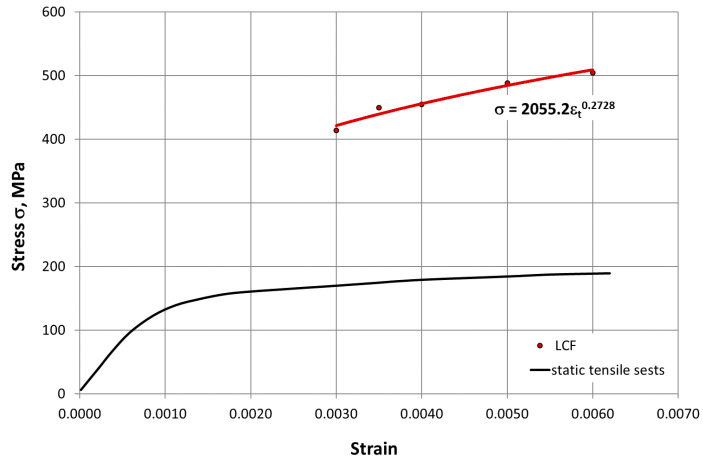
Cyclic strengthening curve for HR6W alloy at 650 °C against a curve determined in a static tensile test (LCF—Low Cycle Fatigue).

The mechanical characteristics of HR6W determined in the low-cycle fatigue tests at room temperature and elevated temperature are summarized in Table 2 and Table 3. Based on these data, cyclic stress–strain curves for this alloy were plotted as presented in Figure 10 and Figure 11, which were described by a mathematical model in the form of $\sigma_{\mathrm{an}}=A\cdot \varepsilon_{\mathrm{ac}}{}^{b}$ and coupled with a curve determined in the static tensile test. 

**Figure 12:**
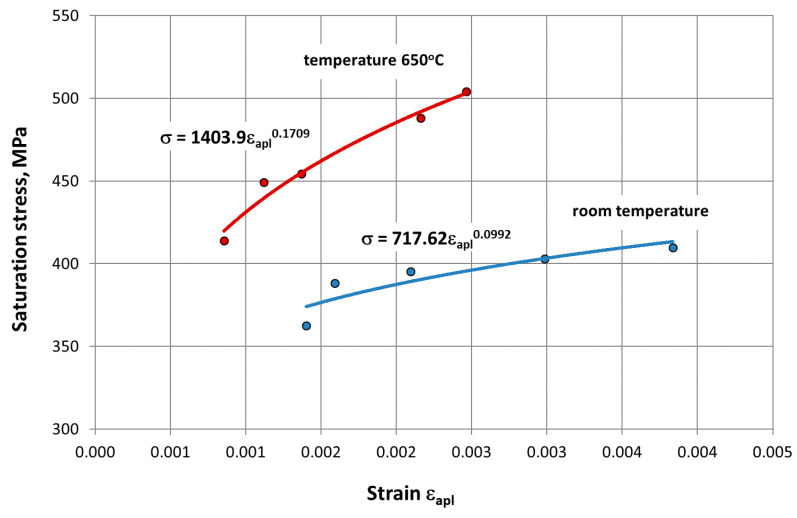
Cyclic strengthening curves for HR6W alloy at room temperature and 650 °C.

As can be seen from the comparison of the curves, the HR6W alloy under the fatigue test conditions was characterized by cyclic hardening both at room temperature and at 650 °C. In addition, mathematical models of cyclic strengthening were developed by $\sigma_{\mathrm{an}}=\;K\prime \cdot \varepsilon_{\mathrm{apl}}{}^{\;n\prime }$ (Figure 12), which were described by the expression, where K′—is the cyclic strength coefficient, and n′—the cyclic weakening coefficient.

The analysis of the characteristics obtained indicates cyclic strengthening of the HR6W alloy both at room temperature and elevated temperatures. At 650 °C, the strengthening process is significantly greater. A greater increase in the value of the stress σ_an_ is visible with the increase in the amplitude of the strain ε_apl_. In this case, the value of the cyclic strength coefficient K’ = 1403.9 MPa and the cyclic weakening coefficient n’ = 0.1709 are significantly higher than those determined for room temperature, i.e., K’ = 717.62 MPa and n’ = 0.0992 (Figure 12). The increase in stresses at room temperature from the initial state to the saturation state was found to be on average 80 MPa, while the temperature of 650 °C was on average 240 MPa.

**Figure 13:**
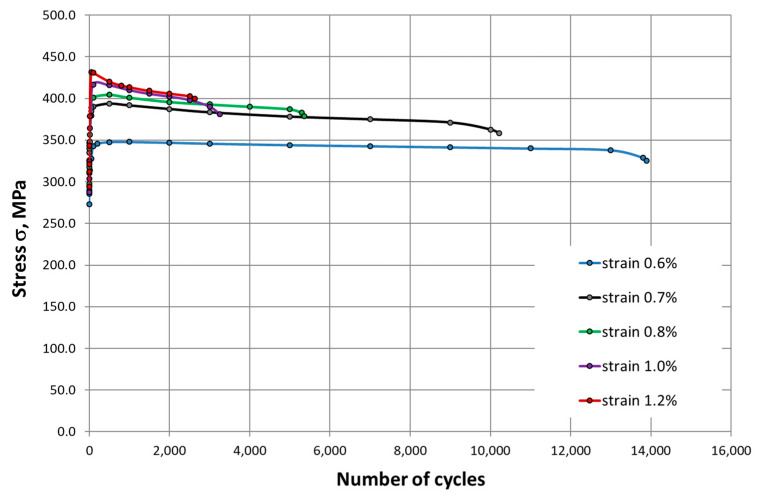
Evolutions of the stress range of HR6W at room temperature.

**Figure 14:**
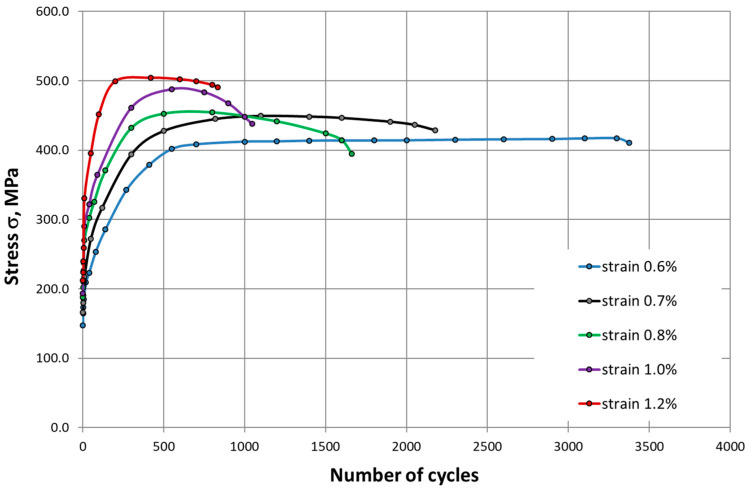
Evolutions of the stress range of HR6W at 650 °C.

Evidence for a much higher intensity of strengthening at 650 °C can also be seen in the characteristics of cyclic deformation (Figure 13 and Figure 14). The process is particularly dynamic in the initial phases of LCF tests, which was observed for all ranges of total strain. The effect observed here is analogous to the characteristics shown in Figure 10 and Figure 11.

**Figure 15:**
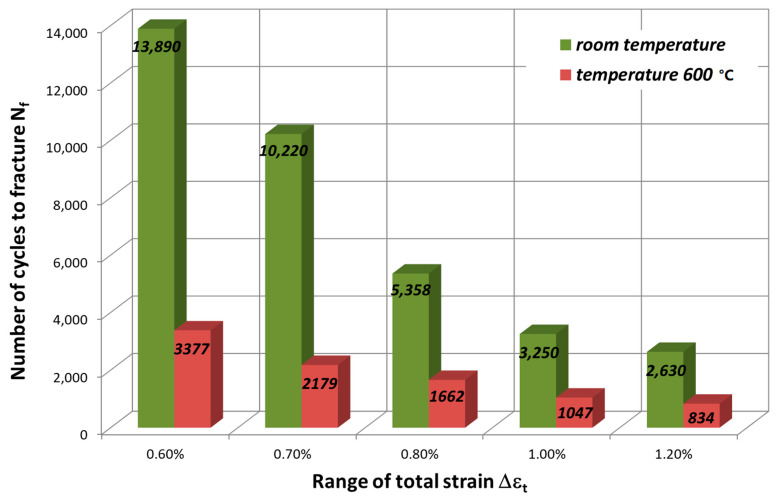
Low-cycle fatigue life N_f_ for HR6W alloy at room temperature and 650 °C.

Based on the results obtained (Table 2 and Table 3), Figure 15 summarizes values for fatigue life N_f_ versus total strain range Δε_t_.

From the analysis of the data, it can be concluded that, at room temperature, a strain-range (Δε_t_) dependent fatigue life N_f_ of the HR6W alloy increased several times (from approx. 3 to approx. 4) as compared to its durability at 650 °C. On the other hand, when analyzing the results of testing the material at 650 °C, it can be noticed that, with a double increase in the total strain range Δε_t_, for example from 0.6% to 1.2%, the number of cycles to failure N_f_ decreased four times from 3377 to 834, and at room temperature up to four times from 13,890 to 2630.

**Figure 16:**
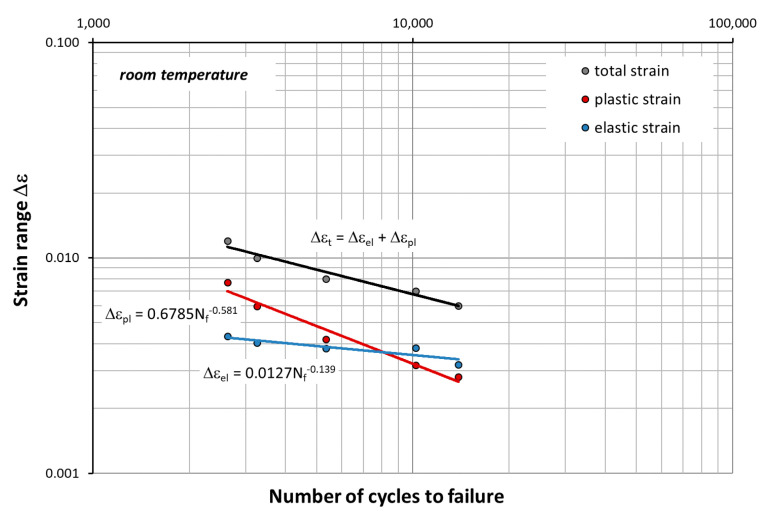
Range strain Δε versus the number of cycles to failure N_f_ at room temperature described by Formula (1).

**Figure 17:**
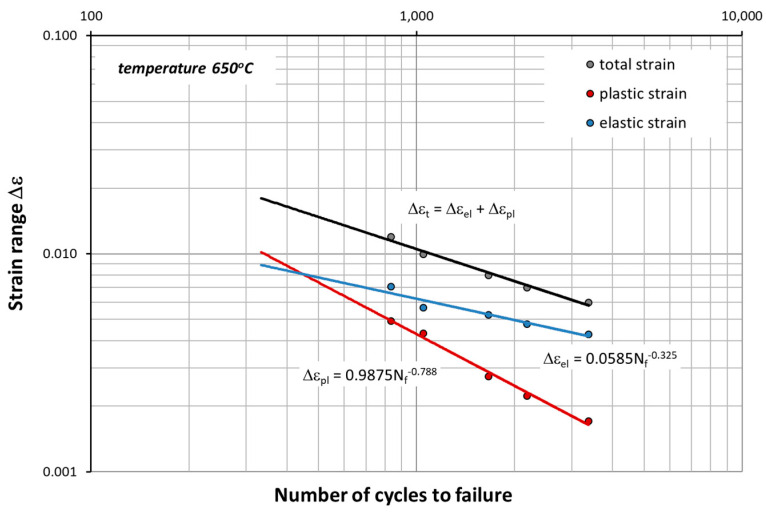
Range strain Δε versus the number of cycles to failure N_f_ at 650 °C described by Formula (1).

Based on the mechanical characteristics in Table 2 and Table 3, the fatigue life graphs for HR6W were also developed (Figure 16 and Figure 17) according to the equation given by Manson–Coffin:

(1) $$\Delta \varepsilon_{t}=\Delta \varepsilon_{pl}+\Delta \varepsilon_{el}=M\cdot N_{f}^{z}+\frac{G}{E}\cdot N_{f}^{\nu }$$

At elevated temperature (650 °C), as shown in Figure 17, in the process of cyclic strain, the elastic component of strain Δε_el_ is dominant. In this case, strain is accompanied by higher stress σ_an_ compared to that occurring at room temperature (Figure 10, Figure 11 and Figure 12). On the other hand, as shown in Figure 11, at room temperature, the cyclic strain occurs with the dominant plastic strain component Δε_pl_. Therefore, it can be assumed that cyclic strain resistance depends mainly on plastic properties.

## 4. Conclusions

On the basis of the results obtained in low-cycle fatigue testing at room temperature and at 650 °C, the following conclusions were formulated:

1. The low-cycle fatigue (LCF) life of HR6W at room temperature, expressed as the number of cycles (N_f_) to fracture, increased several times as compared with its fatigue life at the temperature of 650 °C, depending on the strain range Δε_t_ used in the fatigue tests. The increase is approximately three fold with a greater strain range (Δε_t_ = 1.0 ÷ 1.2%) and approximately four fold with a smaller stain range (Δε_t_ = 0.6 ÷ 0.8%) used in the fatigue tests.
2. Under low-cycle fatigue (LCF) conditions at 650 °C, the fatigue life of HR6W alloy decreased approximately four times from N_f_ = 3377 to 834 cycles, with a double increase in the total strain range from Δε_t_ = 0.6% to 1.2%. On the other hand, at room temperature, the tests showed an approximately 5-fold reduction in fatigue life from N_f_ = 13,890 to 2630 cycles, with a similar increase in total strain range Δε_t_.
3. HR6W alloy shows cyclical strengthening both at room temperature and 650 °C. At elevated temperature, strengthening is significantly greater. It is characterized by a greater increase in stress values σ_an_ along the decrease in strain amplitude ε_apl_. In this case, the value of the cyclic strength factor K’ = 1403.9 MPa and the cyclic strengthening factor n’ = 0.1709 are significantly higher than those determined for room temperature (K’ = 717.62 MPa and n’ = 0.0992, respectively).
4. Analyzing the results of the research on the fatigue of the low-cycle HR6W alloy at the temperature of 650 °C, the process of cyclic hardening of the HR6W alloy is clearly visible. Such behavior of this material is mainly related to the processes of precipitation strengthening taking place in its structure at the boundaries of its grains, in the form of carbides M_23_C_6_.

## Figures and Tables

**Table 1:** Mechanical properties of the HR6W alloy obtained at room temperature and 650 °C.

Mechanical Properties	YS MPa	UTS MPa	A %	Z %
Room temperature	271	624	59	49
650 °C	146	431	70	52

**Table 2:** Mechanical characteristics of HR6W alloy determined in low-cycle fatigue testing at room temperature.

Δε_t_, %	ε_ael_	ε_apl_	σ_an,_ MPa	N_f_
0.6	0.0016	0.0014	362.6	13,890
0.7	0.0019	0.0016	388.4	10,220
0.8	0.0019	0.0021	395.3	5358
1.0	0.0020	0.0030	402.9	3250
1.2	0.0022	0.0038	409.7	2630

where ε_ael_—elastic strain amplitude, ε_apl_—plastic strain amplitude, σ_an_—saturation stress amplitude, N_f_—fatigue life as the number of cycles to fracture.

**Table 3:** Mechanical characteristics of HR6W alloy determined in low-cycle fatigue tests at 650 °C.

Δε_t_, %	ε_ael_	ε_apl_	σ_an,_ MPa	N_f_
0.6	0.00214	0.00086	412.3	3377
0.7	0.00238	0.00112	449.2	2179
0.8	0.00263	0.00137	454.5	1662
1.0	0.00284	0.00216	478.0	1047
1.2	0.00354	0.00246	497.6	834

where ε_ael_—elastic strain amplitude, ε_apl_—plastic strain amplitude, σ_an_—saturation stress amplitude, N_f_—fatigue life as the number of cycles to fracture.

## Data Availability

Data are contained within the article.
